# Radiosensitization of intrahepatic cholangiocarcinoma and metastatic disease in the liver using microbubble cavitation: results from a phase 2 clinical trial

**DOI:** 10.1016/j.canlet.2025.218026

**Published:** 2025-09-15

**Authors:** Corinne E. Wessner, Tania Siu Xiao, Allison Chang, Jenny Liu, Weelic Chong, Ji-Bin Liu, Kristen Bradigan, Flemming Forsberg, Andrej Lyshchik, Patrick O’Kane, Scott W. Keith, Stephen R. Topper, Kevin Anton, John R. Eisenbrey

**Affiliations:** a Department of Radiology, Thomas Jefferson University, Philadelphia, PA, 19107, USA; b School of Biomedical Engineering, Science and Health Systems, Drexel University, Philadelphia, PA, 19104, USA; c Division of Biostatistics and Bioinformatics, Thomas Jefferson University, Philadelphia, PA, 19107, USA

**Keywords:** CEUS=contrast-enhanced ultrasound, ICC=intrahepatic cholangiocarcinoma, MDL=metastatic disease to the liver, mRECIST=modified response evaluation criteria in solid tumors, TARE=transarterial radioembolization, UTMD=ultrasound triggered microbubble destruction, Y90=yttrium-90

## Abstract

Ultrasound triggered microbubble destruction (UTMD) has been shown to sensitize tumors to radiation. This study evaluated the feasibility, safety, and efficacy of using UTMD for radiosensitization in participants with intrahepatic cholangiocarcinoma (ICC) and/or metastatic disease to the liver (MDL) receiving Yttrium-90 transarterial radioembolization (Y90-TARE). This was a prospective single-center IRB-approved clinical trial. Participants received four contrast ultrasound sessions: 1–2 weeks prior to Y90-TARE, 1–4 h post-treatment, and 1 week and 2 weeks post-Y90-TARE. The control group consisted of 1:1 nearest-neighbor propensity score matched historical controls participants that received Y90-TARE as part of standard-of-care. Safety was evaluated through general lab values, physiological monitoring and adverse events. Treatment response was determined by a 1–6 month CT/MRI and evaluated using modified Response Evaluation Criteria in Solid Tumors (mRECIST). Final analysis included 15 participants in each group. There were no differences in general lab values between groups (p > 0.15). In study participants, there were no differences in physiological monitoring between pre and post UTMD sessions (p > 0.20). There was a trend toward a better response distribution in the study group compared to the control group (study group: 0 % progressive disease (0/14), 29 % participants with stable disease (4/14), 21 % participants with partial response (3/14), 50 % participants with complete response (7/14) and control group: 33 % participants with progressive disease (5/15), 27 % with stable disease (4/15), 13 % with partial response (2/15), 27 % with complete response (4/15) when evaluating long-term imaging, p = 0.06). The addition of UTMD in participants that received Y90-TARE was feasible, safe, and might deliver improved treatment response.

## 1. Introduction

The two leading primary liver cancers are hepatocellular carcinoma (HCC) and intrahepatic cholangiocarcinoma (ICC) [1]. The latter is a rarer cancer of cholangiocytes in the liver and accounts for approximately 15 % of all cholangiocarcinoma cases, with mortality rates increasing by 39 % [1–3]. Long-term survival is dismal for ICC patients, with less than 5–10 % surviving five years after diagnosis [3]. Most ICCs have no risk factors associated with the disease and, unfortunately, arise *de novo* [3]. Curative surgical resection is the first line of therapy in patients with ICC without extension beyond the regional lymph nodes and is only possible in less than 30 % of cases with a median disease-free survival of 26 months [3,4].

Outside of primary liver cancers, the liver is one of the most common locations for metastatic disease, mainly due to its dual blood supply [5]. Colorectal cancer (CRC) is the most common primary cancer that will spread to the liver with 30–50 % of cases metastasizing to the liver. Besides CRC other primary cancers that commonly spread to the liver are small cell lung cancer (17 %), non-small cell lung cancer (4 %), cutaneous melanoma (10–20 %), breast (6–38 %), neuroendocrine (20–46 %), gastric (5–40 %) and pancreatic (30–40 %) cases [5,6]. Treatment management for metastatic disease to the liver (MDL) is typically established by multi-disciplinary tumor boards that determine the optimal clinical management. Treatment options for MDL include resection, locoregional therapies, or systemic therapies [7].

Yttrium-90 transarterial radioembolization (Y90-TARE) is a locoregional therapy that has demonstrated some efficacy for treating ICC and MDL [2,8,9]. Currently, TheraSphere (BTG Int., London, UK) and SIR-Spheres (Sirtex Medical Limited, NSW, Australia) are FDA-approved Y90 microspheres (approximately 20–60 μm in diameter) [10]. These beads are delivered to the tumor through a hepatic artery via a catheter, which minimizes treatment to adjacent normal liver tissue and extra-hepatic deposition. Treatment efficacy is typically determined by a 1–6 month contrast-enhanced CT/MRI. In a study evaluating treatment response in 20 patients with ICC, overall objective response rates were 15 % using response evaluation criteria in solid tumors (RECIST) 1.1 and 45 % with the modified response evaluation criteria in solid tumors (mRECIST) [11].

Contrast-enhanced ultrasound (CEUS) is an established imaging technique that uses gas-filled microbubbles with diameters of 1–8 μm [12–14]. Diagnostic CEUS has been shown to be beneficial for a variety of clinical applications including liver imaging and the monitoring of tumor response to radiation therapy [15–19]. A unique advantage of CEUS is the ability to harness bioeffects for therapeutic purposes and generate non-linear oscillations at higher acoustic pressures (>200 kPa) [20–22]. At higher acoustic pressures, ultrasound contrast agents undergo rapid expansion and compression with destruction (e.g. termed ultrasound-triggered microbubble destruction), and the destruction of ultrasound contrast agents have shown to radiosensitize tumors undergoing radiation therapy [23–27]. This concept was confirmed in mechanistic studies showing the combination of UTMD and radiation therapy leads to the vascular shutdown of tumor endothelial cells with an increased accumulation of apoptosis (endothelial cell death) in the tumor, without disrupting normal adjacent tissue [25,28–30].

Ultrasound microbubble-based radiosensitization has been validated in a randomized, phase 2, single-center clinical trial in HCC participants receiving standard of care Y90-TARE (control group) and Y90-TARE with three UTMD sessions (study group) [23,31]. The study demonstrated an improved treatment response in the group who received UTMD compared to the group that received standard of care Y90-TARE [23,31]. Additionally, the study showed that incorporating UTMD is safe and led to an improvement in overall survival improvement in the group that received UTMD [23,31]. However, that study used 2D ultrasound for microbubble destruction and focused solely on HCC patients (whose clinical prognosis is better relative to patients with ICC or MDL). Consequently, the purpose of this pilot clinical trial was to evaluate the safety and preliminary efficacy of volumetric UTMD to sensitize ICC and MDL tumors to Y90-TARE.

## 2. Materials and methods

### 2.1. Study design

This study was a single-center clinical trial conducted at Thomas Jefferson University in Philadelphia, PA, USA. The study was approved by the local Institutional Review Board (#21F.1081) and trial registry at ClinicalTrials.gov listed under NCT #05328167 (https://clinicaltrials.gov/study/NCT05328167) and FDA IND no. 126,768. Recruitment occurred from April 2022 to December 2024. ICC and MDL participants treated with Y90-TARE at our institution are reviewed by a multi-disciplinary liver tumor board that consists of interventional and abdominal radiologists, hepatologists, transplant surgeons, and medical and radiation oncologists. The source of diagnosis for all participants was biopsy. An independent data safety monitoring board evaluated safety for the duration of the study. The study inclusion and exclusion criteria are provided in Table 1.

### 2.2. Study participation

Participants in the study group were enrolled by the principal investigator (JRE) or interventional radiologists (KA and SRT) and provided written informed consent at the time of their pre-procedural flow mapping study. The control group consisted of 1:1 nearest-neighbor propensity score matched historical controls that received Y90-TARE in the past 10 years as part of their standard of care treatment. The participants in the study group received four UTMD sessions. The first ultrasound session with no UTMD imaging, only non-linear CEUS imaging, occurred approximately 2 weeks prior to Y90-TARE procedure. The second UTMD session was performed 1–4 h post Y90-TARE and the third UTMD session occurred approximately 1 week post Y90-TARE. The fourth and final UTMD session occurred appoximately 2 weeks post Y90-TARE. The timing of the second and third UTMD session was chosen partially based on the Y90 half-life of 64 h, and on participant-scheduled appointments and availability.

### 2.3. Procedures

The Y90-TARE procedure involves a mapping flow study typically two weeks prior to treatment. This planning arteriogram identifies arterial supply to the target tumor and surrounding liver parenchyma, assesses for arterial variants that may result in non-target embolization during the planned treatment. Once a patient completes the flow study and is deemed an appropriate candidate for Y90-TARE, dosimetry is performed using the MIRD single compartment model [32]. For ICC and MDL, Y90-TARE is performed using either glass beads (Theraspheres; Boston Scientific, Marlborough, MA, USA) or resin beads (SIR-Spheres, SIRTex, Woburn, Massachusetts, USA).

As part of the experimental ultrasound protocol, the infusion of ultrasound contrast agent consisted of 5 ml of activated Optison (GE HealthCare, Princeton, NJ, USA) mixed in 50 ml saline and infused over a 10-min period at a rate of 120 ml/h through a peripheral arm vein. CEUS examinations were performed by a sonographer or physician (CW and JBL). After confirmation of contrast enhancement, 2D and 3D CEUS imaging was performed with a modified Logiq GE E10 scanner (GE HealthCare, Waukesha, WI, USA) using a RAB6-D probe (frequency 3.3–5.0 MHz and volume rate: 1–4.4 vol/second). For each CEUS examination, participants were asked repeatedly to temporarily halt respiration (for approximately 5–10 s) to acquire 2D and 3D CEUS imaging over the 10-min infusion. For CEUS examination 1, imaging consisted of baseline 2D and 3D CEUS to evaluate pre-treatment tumor enhancement without UTMD. In CEUS examinations 2–4 (post Y90-TARE), UTMD (destructive pulses lasted approximately 4 s in each acquisition) was performed using replenishment imaging at the tumor midline. 2D flash-replenishment was used during the initial period (generally 3–4 cycles), followed by 3D UTMD for the remainder of the contrast infusion. The parameters for 2D and 3D CEUS imaging during UTMD were mechanical index: 0.9 ± 0.29 and intensity spatial peak temporal average (IPSTA) 150.27 ± 24.12 mW/cm^2^ then with low-intensity replenishment imaging mechanical index: 0.16 ± 0.14 and IPSTA 3.16 ± 5.48 mW/cm^2^. The high-intensity UTMD session with low-intensity replenishment sequences were repeated throughout the 10-min infusion.

### 2.4. Treatment outcomes

Treatment efficacy was determined by participant’s 1–6 month contrast-enhanced CT or MRI, which were read by two board-certified abdominal radiologists (AL and POK) with 15 and 20 years experience using mRECIST. The radiologists were blinded to the group assignments and consensus was reached in cases of disagreement. For safety evaluation, changes in general lab values, vital signs pre- and post-UTMD sessions, and adverse events were evaluated. General lab blood values measured were creatinine, total bilirubin, white blood cell count, hemoglobin, aspartate aminotransferase (AST), albumin, alanine aminotransferase (ALT), and alkaline phosphatase (ALP). These values were assessed prior to Y90-TARE and 3 weeks to 3 months post treatment. The timing of the general lab value was determined based on the participant’s clinical care management. For vital signs, temperature, heart rate, systolic and diastolic blood pressure were evaluated immediately before and after each CEUS session. All adverse events (AEs) in the study group were reported and reviewed by the medical monitor (appointed by the Data Safety Monitoring Board). AE occurrence was monitored for 30 min after each CEUS session and delayed AEs were monitored for 30 days. All AEs were graded using Common Terminology Criteria for Adverse Events (CTCAE) [33].

### 2.5. Statistical analysis

Statistical analysis was supervised by the project statistician (SWK) and performed in GraphPad PRISM version 10.1.2 (Dotmatics, Boston, MA, USA) and R version 4.4.1 (R Foundation for Statistical Computing, Vienna, Austria). Sample size for patients receiving UTMD in this pilot study was primarily driven by patient availability and larger, multicenter randomized trials will likely be needed to fully demonstrate efficacy. Fifty-five historical control patients having received Y90-TARE were candidates for 1:1 nearest-neighbor propensity score matching to the study group participants using the matchit package in R. Logistic regression was used to generate the propensity scores as the predicted probabilities of UTMD use conditioned on participant age, ECOG status, chemotherapy use (yes/no), absorbed radiation dose, disease type (ICC, CRC, RCC, or NET), and tumor size. The effect of Y90-TARE plus UTMD on tumor response was tested using a two-sided Mann-Whitney *U* test to determine a difference in mRECIST distributions between groups. For general lab values, the differences from baseline to 3 weeks to 3-months post Y90-TARE were analyzed using unpaired t-tests. General lab values were reported as a mean with standard deviation. For vital signs in the study group, a paired *t*-test was performed using 95 % confidence intervals (CIs). For one-year survival and time to next treatment (TTNT), log-rank (Mantel-Cox) statistical test was used. Tumor specific TTNT was defined as the time from the date of Y90-TARE until the date of tumor-specific locoregional treatment. In terms of follow-up and timings, one-year survival was determined by the date the participant signed informed consent until there was a death from any cause. P-values less than 0.05 were considered nominally statistically significant in this study. Authors had sole control of the data which will be made available upon reasonable request.

## 3. Results

**Fig. 1.**
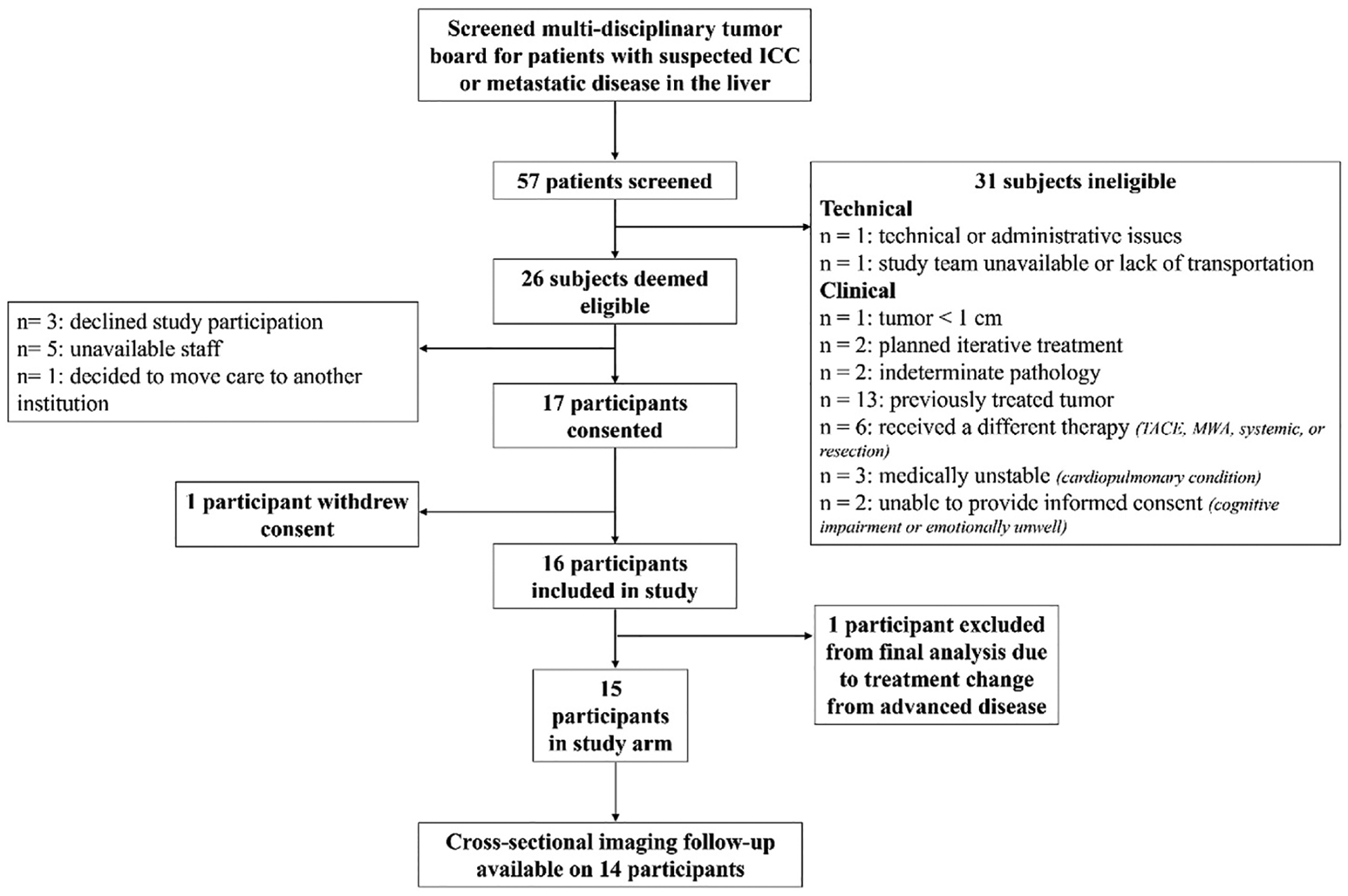
Trial workflow.

**Fig. 2.**
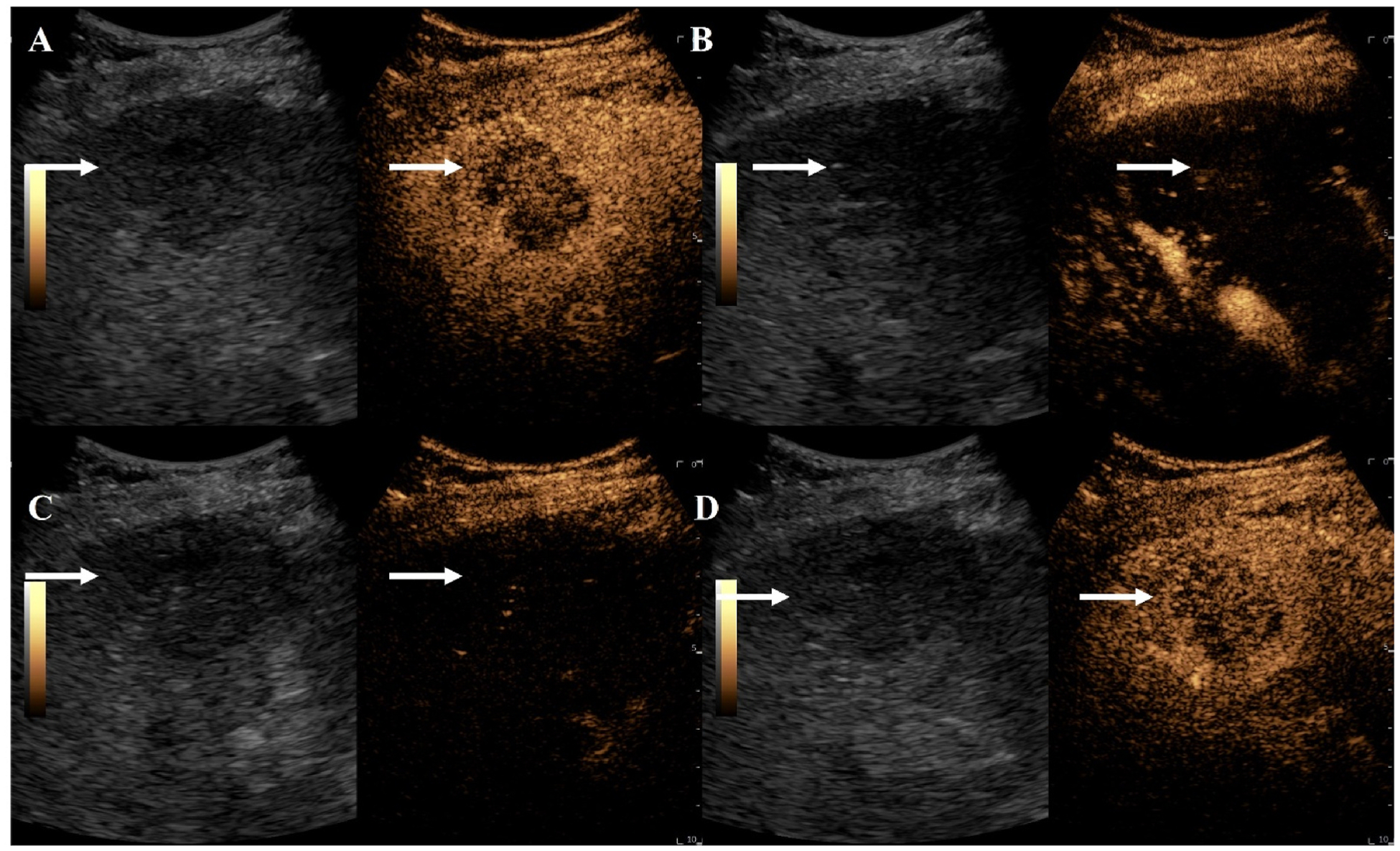
An example of 2D CEUS UTMD imaging. 2D UTMD CEUS imaging of an intrahepatic cholangiocarcinoma participant with a 6.1 cm segment 5/6 tumor 2 weeks post Y90-TARE. (A) Dual non-linear harmonic imaging (right) and B-mode imaging (left) at peak enhancement demonstrating viability within the tumor. (B) An UTMD with high intensity ultrasound pulse applied to the tumor (ISPTA = 165.9). (C) Immediately post UTMD demonstrating almost complete destruction of ultrasound contrast agents within the field of view and (D) reperfusion of ultrasound contrast agents in the tumor demonstrating continued enhancement at 2 weeks post-treatment correlating with stable disease on mRECIST. White arrow indicates the intrahepatic cholangiocarcinoma. UTMD = ultrasound-triggered microbubble destruction, CEUS = contrast-enhanced ultrasound, Y90-TARE = yttrium-90 transarterial radioembolization, ISPTA = intensity spatial peak temporal average, mRECIST = modified response evaluation criteria in solid tumors.

**Fig. 3.**
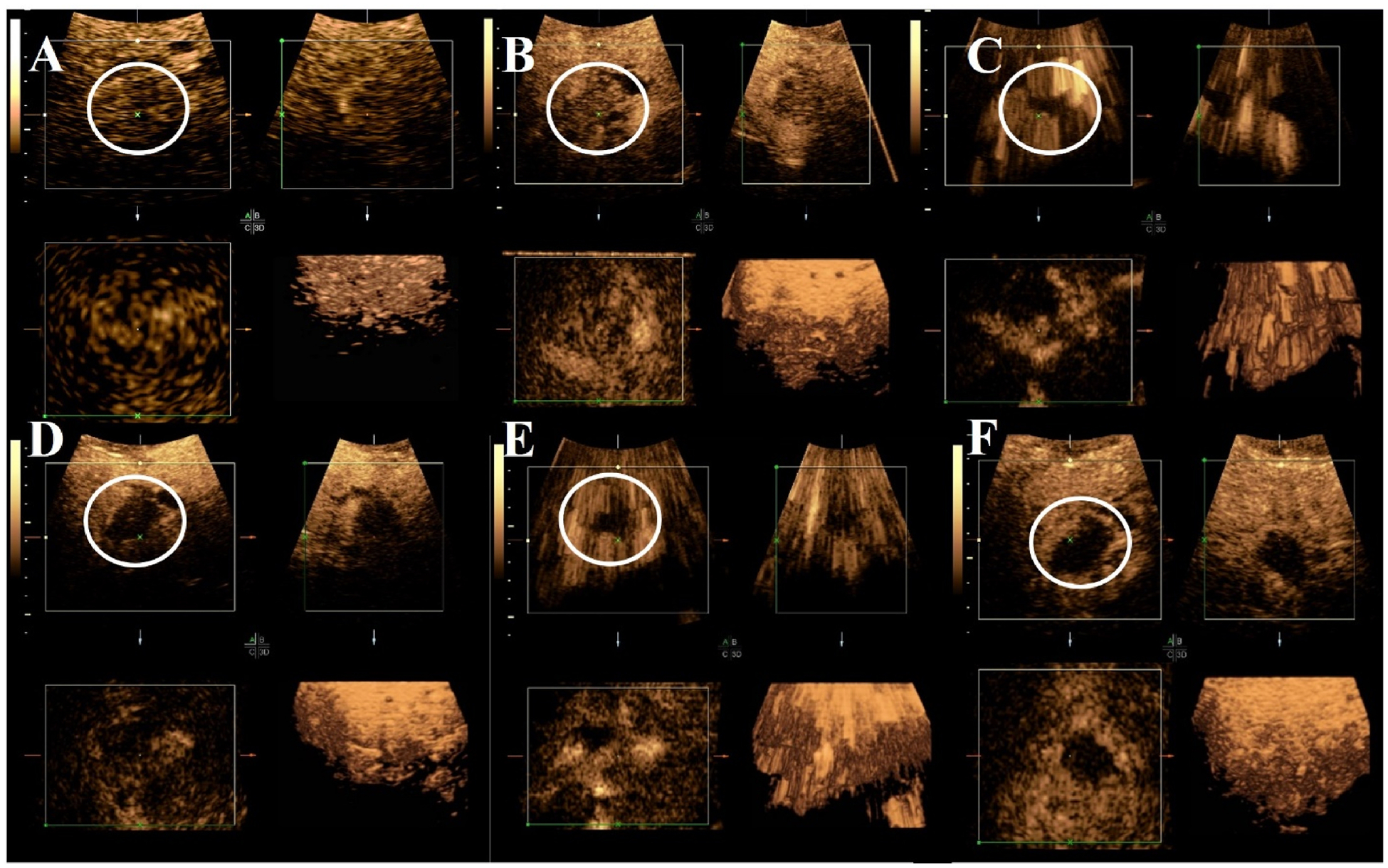
An example of serial 3D UTMD imaging. 3D UTMD CEUS imaging of an intrahepatic cholangiocarcinoma study participant with a 4.1 cm segment 2/3 tumor. Successive 3D CEUS imaging over four timepoints demonstrates (A) complete enhancement two weeks prior to Y90-TARE. (B) CEUS imaging immediately post Y90-TARE with internal enhancement. (C) UTMD high intensity pulse (ISPTA = 176 mW/cm^2^) immediately post Y90-TARE. (D) 1 week post Y90-TARE with minimal contrast enhancement noted within the intrahepatic cholangiocarcinoma. (E) UTMD high intensity pulse (ISPTA = 152.7 mW/cm^2^) at 1 week post Y90-TARE. (F) 2 weeks post Y90-TARE no contrast enhancement is noted within the intrahepatic cholangiocarcinoma, correlating with complete response observed on mRECIST. White circle corresponds to intrahepatic cholangiocarcinoma tumor. UTMD = ultrasound-triggered microbubble destruction, CEUS = contrast-enhanced ultrasound, Y90-TARE = yttrium-90 transarterial radioembolization, ISPTA = intensity spatial peak temporal average, mRECIST = modified response evaluation criteria in solid tumors.

A total of 30 participants are included in the final analysis with 15 study group participants who signed informed consent and 15 historical control participants. Notably, one patient with cholangiocarcinoma was enrolled and underwent baseline CEUS, but their Y90-TARE was aborted following detection of metastatic disease in drained ascites fluid (obtained prior to any ultrasound exam). This patient was subsequently referred for systemic chemotherapy by her clinical team. In the study group, there were 11 participants who completed all four CEUS exams. Four participants completed three out of the four CEUS exams (difficult intravenous catheter insertion n = 1, transportation n = 2, abdominal discomfort n = 1). Fig. 1 demonstrates the study workflow. The mean age of the study group was 66 ± 10.4 years and 67 ± 7.5 years in the control group. Table 2 summarizes participant demographics for both groups. The mean absorbed radiation dose in the study group was 248.7 ± 161.2 Gy while it was 260.9 ± 191.1 Gy in the control group (p = 0.85). For patients with ICC, the average treated tumor size was 3.3 cm (range of 1.1–6.9 cm and a median total number of hepatic tumors of 2 (range of 1–5). For patients with colorectal tumors (n = 2), the treated tumor sizes were 4.0 and 2.8 cm with a total of 2 and 4 total hepatic masses respectively. The patient with metastatic renal cell carcinoma had a solitary 4.8 cm mass. The patient with metastatic pancreatic neuroendocrine carcinoma had a treated tumor size of 2.1 cm and a total of 4 hepatic tumors. Ultrasound contrast was visualized in all participants in the study group. An example of 2D UTMD sessions is shown in Fig. 2 and an example of 3D CEUS over each timepoint with evident pre and post treatment changes is provided in Fig. 3.

Importantly, there were no adverse events (AEs) in the study group related to Optison. There was no statistically significant difference for changes in general lab values from prior to Y90-TARE to approximately 3 weeks to 3 months post-treatment between the study group and control group (p > 0.15). Additionally, there were no statistically significant differences in physiological vital signs pre to post UTMD sessions in the study group (p > 0.20) (Table 3).

**Fig. 4.**
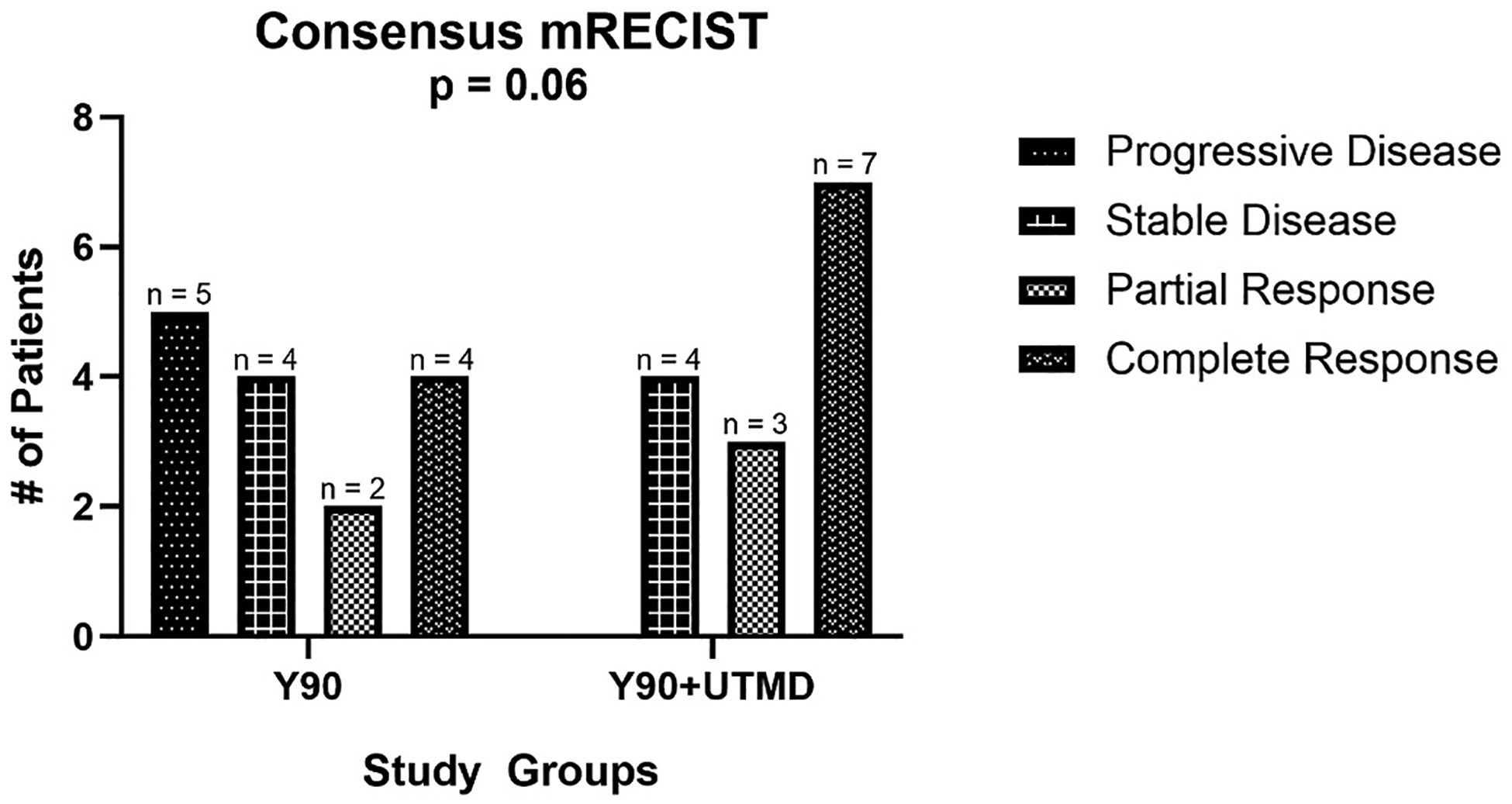
Bar chart of consensus mRECIST in ICC and MDL participants.

In the study group, one participant was excluded from the treatment efficacy dataset, because the participant received re-treatment prior to follow-up imaging. In the study group, 0 % of participants had progressive disease (0/14), 29 % of participants had stable disease (4/14), 21 % of participants had partial response (3/14), and 50 % of participants had complete response (7/14). In the control group, there were 33 % participants with progressive disease (5/15), 27 % with stable disease (4/15), 13 % with partial response (2/15), and 27 % with complete response (4/15). Though these response distributions show a difference in the direction of improved response in patients having UTMD added to Y90-TARE, it was not statistically significant (p = 0.06). Consensus mRECIST for all participants are provided in Fig. 4.

Consensus mRECIST outcomes between the control group (Y90 alone) and study group (Y90 with UTMD) in all participants with both ICC and MDL demonstrating an improved distribution in the response rate in the study group compared to historical controls. However, this was not statistically significant (study group, zero participants had progressive disease (0 %, 0/14), four participants with stable disease (29 %, 4/14), three participants with partial response (21 %, 3/14), and seven participants with complete response (50 %, 7/14) and in the control group, five participants with progressive disease (33 %, 5/15), four with stable disease (27 %, 4/15), two with partial response (13 %, 2/15), and four with complete response (27 %, 4/15), (p = 0.06)).

**Fig. 5.**
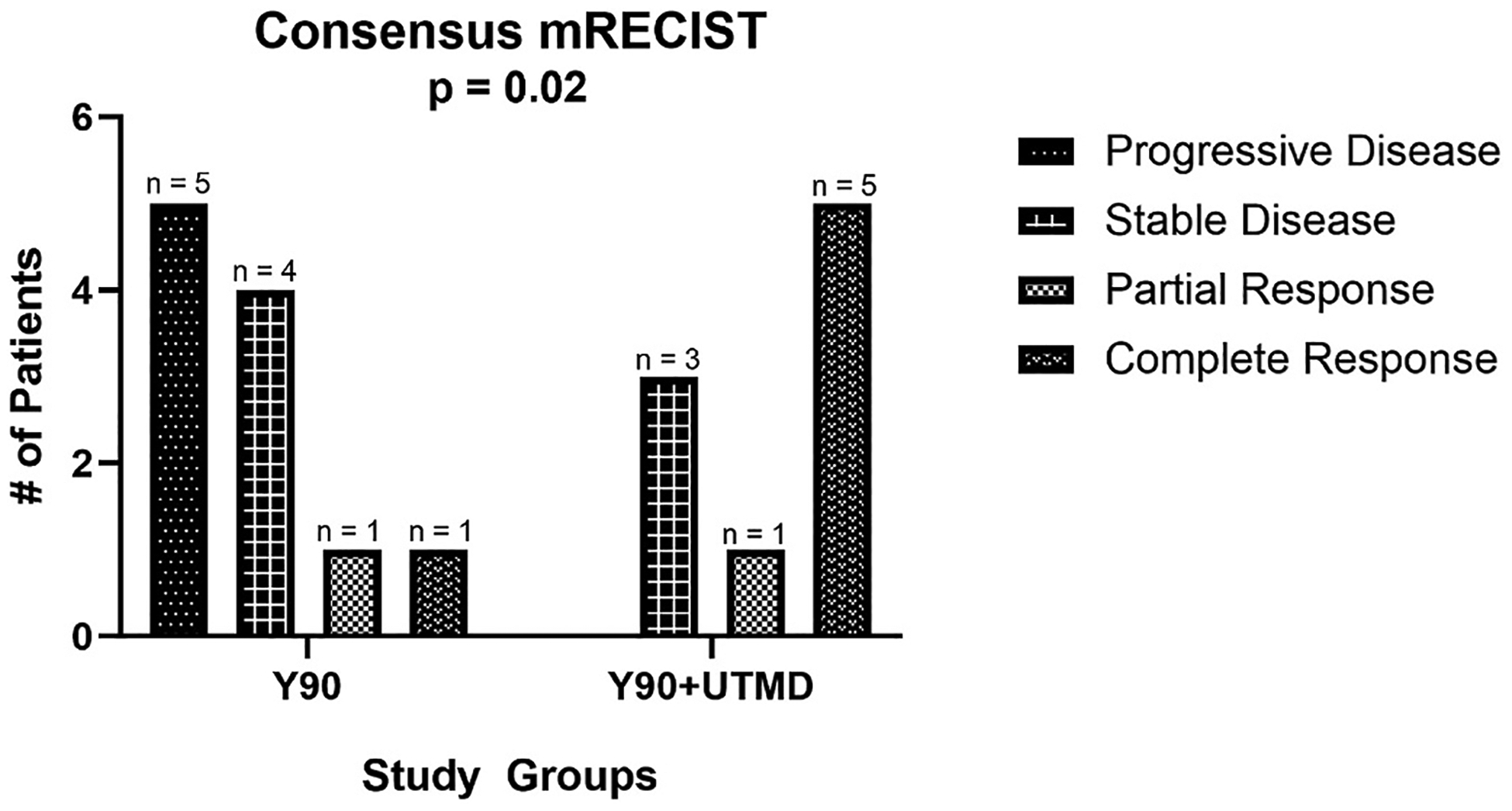
Bar chart of consensus mRECIST in ICC participants.

When evaluating the subgroup of ICC participants (Table 4), there was a statistically significant difference in their distributions of response favoring the study group compared to control group (p = 0.02). In the ICC study group, there were 0 % participants with progressive disease (0/9), 33 % participants with stable disease (3/9), 11 % participant with partial response (1/9), and 56 % participants with complete response (5/9). In the control group, there were 45 % participants with progressive disease (5/11), 36 % participants with stable disease (4/11), 9 % participant with partial response (1/11), and 9 % participant with complete response (1/11). Consensus mRECIST for ICC participants in the study and control groups are provided in Fig. 5.

Consensus mRECIST outcomes between the historical control group (Y90 alone) and study group (Y90 with UTMD) in only the ICC participants showing an improvement in distribution of response rate in the study group compared to the control group (in study group there were zero participants with progressive disease (0 %, 0/9), three participants with stable disease (33 %, 3/9), one participant with partial response (11 %, 1/9), and five participants with complete response (56 %, 5/9) and in control group, there were five participants with progressive disease (50 %, 5/10), 4 participants with stable disease (40 %, 4/10), one participant with partial response (10 %, 1/10), and one participant with complete response (10 %, 1/10), p = 0.02).

When comparing one-year survival (Table 4), there were 4 (27 %, 4/15) deaths in the study group and 4 (27 %, 4/15) deaths in the control group (p = 0.81). Additionally, the one-year survival hazard ratio was 0.84 (95 % CI: 0.21 to 3.39). Similarly, no statistically significant difference in TTNT was observed (p = 0.10).

## 4. Discussion

This single center clinical trial showed that incorporating UTMD into the Y90-TARE treatment of ICC and MDL tumors is feasible, safe and showed some evidence of improved response relative to matched historical controls. Importantly, there were no AEs relating to the ultrasound contrast agent Optison in the study group and no statistically significant differences in general lab values between the study and control groups.

Currently, limited research exists on treatment outcomes for patients with ICC and MDL that receive Y90-TARE. In a study of 29 patients with unresectable ICC treated with Y90-TARE, there was a 12 % objective response rate using RECIST 1.1, while disease control rate was 73 % [34]. However, another study consisting of 28 patients with localized ICC treated with Y90-TARE only reported a cumulative radiologic response rate of 57 % [35]. In MDL tumors, one study evaluated 49 patients with metastatic neuroendocrine tumors to the liver demonstrated 53 % of partial response rate using RECIST 1.1 [9]. Importantly, there is a need for additional drug delivery therapies to improve this wide response rate in ICC and MDL patients.

In this trial when evaluating both ICC and MDL tumors, the study group had improved response rates compared to the control groups, however, this was not statistically significant. Since ICC and MDL tumors have different tumor microenvironments and etiologies, we evaluated treatment responses between tumor cohorts separately. Although based on a small sample size (study group: n = 9 and control group: n = 11), ICC tumors had a statistically significant improvement in response rates compared to historical controls. These improvements in radiosensitization are consistent with recent emerging reports using microbubble cavitation to augment radiotherapy in recent phase I and II clinical trials [26,27,31]. These trials span both external beam radiation in the treatment of head and neck cancer [26] and breast cancer [27], as well as a recently completed randomized trial in Y90-TARE of HCC [31]. Results from this study are consistent with these reports, demonstrating both safety and efficacy of using ultrasound-stimulated microbubbles with radiotherapy.

In our study we used the mRECIST criteria to evaluate tumor response on follow-up CT/MRI, similar to many others, including several phase II trials in intermediate-stage liver tumors using mRECIST endpoints because of its focus on viable tumor. [36,37]. The main rationale is that mRECIST focuses on residual enhancing tumor rather than total lesion size, as in RECIST 1.1, which helps distinguish necrotic or fibrotic tissue commonly occurring in treated tumors from actively perfusing cancer. We acknowledge the limitations of mRECIST for ICC and certain metastases where in some rare cases, mRECIST may classify a partially treated lesion as complete responder even if a viable fibrotic mass persists. However, we believe that mRECIST is preferable to conventional RECIST 1.1 in our patient population, since a much greater number of nonviable tumors are replaced with scar tissue after locoregional treatment, resulting in misleading “partial response” or “stable disease” categorization with RECIST 1.1. Additionally, a variety of biomarkers have been proposed for the detection and treatment response monitoring of both ICC and MDL [38–40]. These tests include circulating tumor DNA, non-coding RNA, extracellular vesicles, cytokines, and a variety of metabolites [38]. While these biomarkers are not yet part of clinical care, in the future, a multiparametric approach that combines cross sectional imaging and systemic biomarkers will likely better evaluate locoregional treatment response in these challenging tumors.

In terms of one-year time to next treatment, there was no significant difference between groups. There are many factors that could contribute to the multi-disciplinary tumor board decision for retreatment. However, in this patient population the clinical management has become increasingly aggressive given the emerging data of Y90-TARE and since these patients are not surgical candidates.

Future work will focus on incorporating UTMD augmented radiosensitization for ICC and MDL in larger, randomized clinical trials. Since the response rates are broad with ICC and MDL, additional imaging modalities, other than MRI or CT, should be explored. Currently, there is limited research on predicting and monitoring treatment response using CEUS in patients that receive Y90-TARE [41].

There are several limitations to this trial. While we demonstrated that 3D UTMD is feasible and safe for augmenting Y90-TARE in patients with ICC and MDL, larger studies are needed to demonstrate its effectiveness. Given the different tumor types in this study, this may limit the effectiveness of microbubble-based radiosensitization. Finally, the historical control group was collected from a ten-year period. While all participants received Y90-TARE, and we demonstrated similar absorbed radiation doses across populations, clinical management could potentially have changed over that period, particularly regarding decisions to retreat.

In conclusion, this study showed that incorporating UTMD in ICC and MDL participants that received Y90-TARE was safe and may have the potential to lead to improved response rates. Future work will focus on larger, multi-center clinical trials and incorporating UTMD augmented therapies into other malignancies.

## Figures and Tables

**Table 1:** Study criteria.

**Inclusion Criteria:**
Be scheduled for sub-lobar radioembolization therapy of a previously untreated intrahepatic cholangiocarcinoma or liver metastasis greater than 1 cm but small enough to be fully visualized in the ultrasound three-dimensional (3D) volume (approximately 6 cm maximum diameter, but depth dependent)
Be at least 18 years of age
Be medically stable
If a female of child-bearing age, have a negative pregnancy test prior to each ultrasound exam
Have signed Informed Consent to participate in the study
**Exclusion Criteria:**
Females who are pregnant or nursing
Patients with recent cerebral hemorrhage
Patients with known sensitivities to albumin, blood, or blood products
Patients with known hypersensitivity to perflutren
Patients with known congenital heart defects
Patients with severe emphysema, pulmonary vasculitis, or a history of pulmonary emboli
Patients with bilirubin levels >2 mg/dL

**Table 2:** Participant demographics.

Parameter	Control group	Study group
**Participant**, n	n = 15	n = 15
**Sex**, n (%)		
Male	8 (53 %)	8 (53 %)
Female	7 (47 %)	7 (47 %)
**Age (years)**, mean ± SD	67 ± 7.5	66 ± 10.4
**Clinical Presentation**, (mean ± SD)		
Body mass index (kg/m^2^)	25.6 ± 4.7	27.3 ± 4.7
Treated tumor size (cm)	4.3 ± 3.1	3.3 ± 1.7
Absorbed Radiation Dose (Gy)	260.9 ± 191.1	248.7 ± 161.2
ECOG performance score, n (%)		
0	0 (0 %)	1 (7 %)
1	13 (87 %)	12 (80 %)
2	2 (13 %)	2 (13 %)
**Tumor Type, n (%)**		
Intrahepatic cholangiocarcinoma	11 (73 %)	10 (67 %)
Metastatic disease to the liver	4 (27 %)	5 (33 %)
Colorectal carcinoma	2 (13 %)	3 (20 %)
Renal cell carcinoma	1 (7 %)	1 [7]%
Pancreatic neuroendocrine carcinoma	1 (7 %)	1 (7 %)

n = number of participant, ECOG = Eastern Cooperative Oncology Group.

**Table 3:** Clinical presentation.

Parameter	Control group	Study group	*Difference	p value
**Adverse Events**, n (%)	N/A	0, (0.0 %)	N/A	N/A
CTCAE Grades, n (%)	N/A	0, (0.0 %)	N/A	N/A
**Changes in general lab values (mean ± standard deviation)**				
AST (IU/L)	3.33 ± 16.28	4.93 ± 14.46	−1.60 (−13.12, 9.92)	0.78
ALT (IU/L)	2.60 ± 17.49	−0.93 ± 18.32	3.53 (−9.86,16.93)	0.59
ALP (IU/L)	44.20 ± 69.39	37.00 ± 58.08	7.20 (−40.66, 55.06)	0.76
Albumin (g/dL)	−0.37 ± 0.47	−0.28 ± 0.31	−0.09 (−0.38,0.21)	0.55
Hemoglobin (g/dL)	−0.31 ± 0.82	−0.83 ± 3.21	0.51 (−1.24, 2.27)	0.55
White blood cell count (10^9^/L)	3.85 ± 10.35	−0.14 ± 2.36	3.99 (−1.62, 9.61)	0.15
Creatinine (mg/dL)	0.08 ± 0.57	−0.05 ± 0.25	0.13 (−0.19, 0.46)	0.42
Total bilirubin (mg/dL)	0.15 ± 0.38	0.12 ± 0.41	0.03 (−0.27, 0.32)	0.85
**Changes in vital signs (study group only), mean (95 % CI)**				
Temperature (°F)	N/A	0.17 (−0.10, 0.45)	N/A	0.20
Heart rate (beats/min)	N/A	−0.53 (−3.54, 2.48)	N/A	0.71
Systolic blood pressure (mmHg)	N/A	−2.07 (−11.16, 7.03)	N/A	0.63
Diastolic blood pressure (mmHg)	N/A	−1.0 (−5.12, 3.12)	N/A	0.61

n = number of participants, CTCAE= Common Terminology Criteria for Adverse Events, AST = aspartate aminotransferase, ALT = alanine aminotransferase, ALP = alkaline phosphatase, N/A = not applicable,

* Difference: Treatment effects were estimated as differences in proportions or means and shown with 95 % CI, Changes in general lab values are reported as the change from baseline to 3 weeks to 3 months post treatment, Changes in the vital signs is reported on for the study group and it is the difference from post to pre UTMD session.

**Table 4:** Treatment outcomes.

	Control group	Study group	p-value
**Overall mRECIST**, n (%)			0.06
Progressive Disease	5 (33 %)	0 (0 %)	
Stable Disease	4 (27 %)	4 (29 %)	
Partial Response	2 (13 %)	3 (21 %)	
Complete Response	4 (27 %)	7 (50 %)	
**ICC mRECIST**, n (%)			0.02
Progressive Disease	5 (45 %)	0 (0 %)	
Stable Disease	4 (36 %)	3 (33 %)	
Partial Response	1 (9 %)	1 (11 %)	
Complete Response	1 (9 %)	5 (56 %)	
**MDL mRECIST, n (%)**			0.37
Progressive Disease	0 (0 %)	0 (0 %)	
Stable Disease	0 (0 %)	1 (20 %)	
Partial Response	1 (25 %)	2 (40 %)	
Complete Response	3 (75 %)	2 (40 %)	
**One-year time to next treatment**			0.10
Events (n (%))	2 (13 %)	6 (40 %)	
Median TTNT (days)	Not reached	Not reached	
Hazard ratio (95 % CI)	0.29 (0.07, 1.15)		
**One-year survival**			0.81
Events (n (%))	4 (27 %)	4 (27 %)	
Median survival (days)	Not reached	Not reached	
Hazard ratio (95 % CI)	0.84 (0.21, 3.39)		

n = number of participants, TTNT = time to next treatment, mRECIST = modified response evaluation criteria in solid tumors.

## Data Availability

Authors had sole control of the data which will be made available upon reasonable request.
